# Severe Diarrhea Outbreaks in Newborn Piglets in China Associated With Porcine Rotavirus B

**DOI:** 10.1155/tbed/5588912

**Published:** 2025-11-21

**Authors:** Mingkai Sun, Tianzeng Li, Wugang Liu, Zhixing Guo, Zunbao Wang, Jianfeng Jiang, Qingxian Li, Biao He, Yidi Guo, Wenjie Gong

**Affiliations:** ^1^State Key Laboratory for Diagnosis and Treatment of Severe Zoonotic Infectious Diseases, Key Laboratory for Zoonosis Research of the Ministry of Education, College of Veterinary Medicine, Jilin University, Changchun, China; ^2^State Key Laboratory of Pathogen and Biosecurity, Changchun Veterinary Research Institute, Chinese Academy of Agricultural Sciences, China; ^3^TECON Biopharmaceutical Co. Ltd., Urumqi, China; ^4^Zhejiang Meibaolong Biotechnology Co. Ltd., Hangzhou, China

**Keywords:** genotyping, phylogenetic analysis, piglet diarrhea, PoRVB

## Abstract

Rotavirus B (RVB) is among the enteric pathogens that can cause gastroenteritis in humans and various animals. However, severe diarrhea in newborn piglets has rarely been reported to be linked to porcine RVB (PoRVB). From 2023 to 2024, outbreaks of newborn piglet diarrhea with 20%–50% morbidity and 4%–10% mortality occurred in three herds situated in Anhui, Liaoning, and Jilin provinces of China. Notably, all samples from these herds tested negative for porcine epidemic diarrhea virus (PEDV), porcine deltacoronavirus (PDCoV), PoRVA and transmissible gastroenteritis virus (TGEV). To identify the causative pathogens of the severe diarrhea in newborn piglets, high-throughput sequencing (HTS) was performed on pooled and individual samples collected from each farm. The results revealed that there were 783,546–1,237,231 mammalian virus reads per pooled sample, with PoRVB being the overwhelmingly dominant virus. A high prevalence of PoRVB (83.3%–100%) was detected in all the affected farms, indicating the association between PoRVB strains and the outbreak of severe piglet diarrhea in the sampled farms. Analysis of *de novo* assembled whole genomes from individual samples revealed that the PoRVB strains AHLW1/2023, JLCG2/2024, and LNDC5/2024 exhibited 81.8%−95.6% nucleotide and 88.9%−99.0% amino acid sequence identities of all 11 gene segments when compared to the most similar reference PoRVB strains. The genotype constellation of AHLW1/2023 was assigned to G34-P[4]-I13-R4-C4-M7-A8-N10-T4-E4-H7, which differs from JLCG2/2024 by one genotype (R7) and from LNDC5/2024 by three genotypes (G16, R7, and M4). This study demonstrated that PoRVB is the primary etiological agent responsible for severe diarrheal outbreaks in newborn piglets. It also highlights the importance of conducting continuous surveillance to effectively control PoRVB infections.

## 1. Introduction

Rotavirus (RV) is a group of enteric pathogens classified under the genus *Rotavirus* within the family *Sedoreoviridae*. It is a significant cause of severe gastroenteritis in children and young animals worldwide [1–5]. These nonenveloped dsRNA viruses have an 11-segmented genome that encodes six structural proteins (VP1–VP4, VP6, and VP7) and five nonstructural proteins (NSP1–NSP5). The viral particles feature a triple-layered capsid structure, consisting of three concentric protein layers [6]. From a taxonomic perspective, the International Committee on Taxonomy of Viruses (ICTV) has identified nine RV species, namely RVA–RVD and RVF–RVJ [7]. These species are distinguished based on the antigenic and genetic divergence of VP6, with a threshold of less than 53% amino acid identity [8]. In terms of genotyping, the Rotavirus Classification Working Group (RCWG) system uses the standardized nomenclature Gx-P[x]-Ix-Rx-Cx-Mx-Ax-Nx-Tx-Ex-Hx to designate the 11 gene segments [9, 10].

Among the RV species that infect humans and pigs (RVA, RVB, RVC, and RVH), RVA is the most clinically significant, primarily because of its global prevalence and association with severe dehydrating diarrhea [2, 11]. Notably, RVF was once thought to be exclusive to avian species [12]. However, it was recently and unexpectedly identified in stunted pigs [13], marking the first time it had been detected in a mammalian host. With the exception of RVA, the epidemiology and pathogenicity of the remaining RVs in pigs remain poorly understood, largely due to the difficulties in isolating these viruses *in vitro* and the complexity of co-infections. RVB was first identified in 1983 during a large-scale adult diarrhea outbreak in China and subsequently in diarrheic suckling piglets in the United States [14, 15]. To date, RVB has been reported to have a broad host range, infecting humans, pigs, cows, sheep, horses, and rats [5, 16–19]. Porcine RVB (PoRVB) infections have been reported in many countries worldwide, while most cases are sporadic, with limited prevalence [20–32]. Although high detection rates (25.9%−46.8%) of PoRVB have been reported in Japanese, American, and Brazilian pig herds, most PoRVB-positive samples were also positive for other diarrhea-associated viruses, such as PoRVA, PoRVC, and PoRVH. This has hindered the establishment of the etiological and pathogenic role of PoRVB strains in piglet diarrhea [33–35].

To date, only three cases of severe diarrhea in newborn piglets have been reported to be associated with PoRVB: (1) In the Buryat Republic, Russia, an intestinal disease outbreak affected 3–5-day-old suckling piglets on a farm. The morbidity and mortality rates were 60% and 8%, respectively. Through next-generation sequencing (NGS), only PoRVB was detected [20]; (2) In the central-western region of Brazil, PoRVB-associated diarrhea occurred in piglets less than 20 days old (with a positive rate of 71.1%) across eight pig farms. The morbidity and mortality rates ranged from 35% to 50% and 10%–50%, respectively. Additionally, co-infection of PoRVB with PoRVH or PoRVA was detected in 6.66% of the samples [26]; (3) In Henan province, China, a diarrhea outbreak affected piglets within 7 days of age on a farm, leading to morbidity and mortality rates of approximately 44.9% and 3.8%, respectively. The identified PoRVB strain was found to be the result of reassortment between strains from herbivores and pigs [25]. Currently, viral diarrhea is one of the most devastating infectious diseases for the pig industry in China. However, during the routine diagnosis of diarrheal viruses, PoRVB has received far less attention compared to porcine epidemic diarrhea virus (PEDV) and PoRVA. As a result, limited information is available regarding the epidemiological situation of PoRVB.

In this study, diarrheic samples were collected from three pig farms experiencing severe piglet diarrhea. RT-PCR detection revealed that these samples were negative for the common diarrheal viruses, including PEDV, transmissible gastroenteritis virus (TGEV), porcine deltacoronavirus (PDCoV), and PoRVA. Subsequently, high-throughput sequencing (HTS) was conducted using pooled samples collected from each farm, along with selective individual samples. Unexpectedly, PoRVB was identified in high abundance as the primary or sole virus present in the diarrheic samples. These findings strongly suggest that PoRVB is implicated in the outbreak of pig diarrhea at the sampled farms. To effectively prevent and control PoRVB-related diarrhea, it is imperative to implement comprehensive and continuous surveillance of this virus. This ongoing monitoring will facilitate early detection, timely intervention, and better management of PoRVB infections within the swine population.

## 2. Materials and Methods

### 2.1. Clinical Samples

Between 2023 and 2024, outbreaks of severe diarrhea in newborn piglets were reported in three distinct provinces in China. In the period from April to June 2023, a large-scale piglet diarrhea outbreak occurred on a pig farm (AHLW) in Anhui province. During this time, at least 13,000 piglets were born, and 30% of the newborn piglets developed severe diarrhea, leading to a mortality rate of about 10% among the affected piglets. In June 2023, six anal swab samples were collected from 3-days-old diarrheic piglets. From January to March 2024, an outbreak of severe and acute diarrhea affected 5–14-day-old piglets on a farm (JLCG) in Jilin province. Over 7000 piglets were born during this period, with approximately 50% showing diarrhea symptoms. The mortality rate was about 4%. The diarrhea pigs exhibited signs of depression and severe dehydration, and excreted yellow, cheese-like feces. After rehydration and drug treatment, 80% of the sick piglets recovered. On the JLCG farm, the back-up sows of diarrheic piglets were vaccinated with a PEDV–TGEV–PoRVA triple live attenuated vaccine 30 days before farrowing and a PEDV–TGEV double inactivated vaccine 15 days before farrowing. Notably, none of the gilts showed diarrhea symptoms. A total of eight anal swab samples were collected from 5–7-day-old diarrheic piglets. In May 2024, piglet diarrhea occurred on a farm (LNDC) in Liaoning province. The farm had 1200 piglets, and the morbidity and mortality rates of piglet diarrhea were 20% and 5%, respectively. The affected piglets excreted grayish-white, thin, and watery feces, and 22 anal swab samples were collected from 5–7-day-old diarrheic piglets, along with five anal swab samples from healthy piglets and two from recovered piglets. During gestation, the diarrheic piglet-derived gilts were vaccinated with live attenuated and inactivated PEDV/TGEV binary vaccines (Jinhe Biological Ltd., Hangzhou, China), yet no diarrhea was observed in these gilts. The diarrheic piglets excreted grayish-white watery feces. All the collected samples were transported to our laboratory using dry ice and subsequently stored at −80°C until further analysis.

### 2.2. Detection of PEDV, TGEV, PDCoV, and PoRVA by RT-nested PCR (nPCR)

The collected fecal swab samples were each added to 1 mL of minimal essential medium (MEM). After incubation at 4°C for 1 h, 200 μL of the leachate was subjected to total RNA extraction using RaPure Viral RNA/DNA Kit (Magen, China). Subsequently, the obtained total RNA was reverse transcribed using M-MLV reverse transcriptase (TaKaRa, China) to synthesize the first-strand cDNA, which served as the template for nPCR to detect major piglet diarrhea-associated viruses, namely PEDV, TGEV, PDCoV, and PoRVA [25]. The amplified PCR products were analyzed via 1% agarose gel electrophoresis.

### 2.3. Viral Metagenomics

To identify potential pathogens causing piglet diarrhea outbreaks, Illumina MiSeq-based metagenomic sequencing was conducted with the pooled diarrheic samples from each pig farm. For meta-transcriptomics (MTT), the total RNA in the pooled or individual samples was first extracted using Trizol reagent (Invitrogen, USA), in which the rRNA was depleted as described previously. The remaining RNA was precipitated with ethanol. RNA libraries were then constructed according to the methodology described previously [36] and subjected to HTS on the Illumina NovaSeq platform (Novogene, Tianjin, China). Regarding multiple displacement amplification (MDA), the samples were processed with the DNeasy Blood & Tissue Kit (QIAGEN GmbH, Germany) to isolate total DNA and RNA. The DNA fraction was amplified using the GenomiPhi V2 DNA Amplification Kit (Cytiva, UK) for DNA library construction. The concentration of the amplified DNA was measured using the Qubit 1x dsDNA HS Assay Kit (Invitrogen, USA). Once the DNA concentration met the sequencing requirements, the DNA library underwent HTS following the same procedures as above at Novogene (Tianjin, China). For each RNA or DNA library, 6 Gb of HTS data were generated. After sequencing quality control, porcine genome sequence reads were first removed using BWA software [37]. The remaining reads were annotated with Diamond [38] and then aligned against the NCBI's non-redundant database with a maximum e-value set as 1e − 5. Viral reads were *de novo* assembled into contigs using Megahit v1.2.9 [39], and the resulting contigs were further verified using the BLAST module of the NCBI online database. To comprehensively assess the presence of bacteria, archaea, and parasites in the samples, the obtained HTS data were analyzed using Metaphlan 4.0.6 software [40].

### 2.4. Validation of Viruses in Clinical Samples by RT-nPCR and nPCR

To determine the positive rates of PoRVB in the diarrheic samples, the cDNA obtained in Section 2.2 was served as the template for nPCR detection. Specific primers were designed using Primer 5.0 software, based on the obtained VP6 sequences of PoRVB (Table 1). Following analysis via 1% agarose gel electrophoresis, the positive PCR products were sent to Sangon Biotech (Shanghai, China) for sequencing and further confirmed by BLAST. Subsequently, positive individual samples were randomly selected for HTS to profile the viral composition associated with diarrhea and to obtain the whole genome sequences of PoRVB strains. Moreover, the presence of other mammalian viruses identified through viral metagenomics, including PoRVA, porcine kobuvirus (PKV), sapovirus (SaV), porcine astrovirus 4 (PAstV4) and porcine circovirus type 3 (PCV3), was also investigated by RT-nPCR and nPCR using specific primers listed in Table 1.

### 2.5. Detection of PoRVB Using RT-Quantitative PCR (RT-qPCR)

To construct recombinant plasmids for establishing a standard curve, a 104-bp fragment of the PoRVB VP6 gene was amplified using 2x Rapid Taq Master Mix (Vazyme, China) with a specific primer pair: RVB-VP6-F (5′ -GACCAGTGAGAGCAATAGAAAG-3′) and RVB-VP6-R (5′-GTTTGCRACTGAAATTGGGGAT-3′). The amplicon was cloned into the pMD-18T vector (TaKaRa, China) following the manufacturer's instructions to generate the recombinant plasmid pMD-PoRVB, which was then prepared as a tenfold serial dilution gradient to serve as a standard for subsequent absolute quantification. Total RNA was extracted from each anal swab using the RaPure Viral RNA/DNA Kit (Magen, China) as mentioned above. Subsequently, RNA quantification was performed with a NanoDrop One Spectrophotometer (Thermo Scientific, USA). Following this, a total of 30 ng RNA was aliquoted from each quantified sample, and reverse transcription was conducted to synthesize cDNA. This reaction was prepared in a 50 μL reaction system using M-MLV reverse transcriptase (TaKaRa, China). RT-qPCR was performed using TB Green Premix Ex Taq (TaKaRa, China) on an Agilent Stratagene Mx3000P Real-Time PCR System (Agilent Technologies, USA). The 20 μL reaction system consisted of 10 μL TB Green Premix Ex Taq (TaKaRa, China), 0.8 μL of each forward and reverse primer, 1 μL cDNA, and 7.4 μL nuclease-free water. The cycling program was 95°C for 2 min, followed by 40 cycles of 95°C for 10 s and 60°C for 20 s. Copy numbers of PoRVB in samples were calculated based on the standard curve generated from the serial dilutions of the recombinant plasmid.

### 2.6. Sequence Comparison and Phylogenetic Analysis

To further elucidate the genetic relationships among the identified PoRVB strains, the gene segments of these strains were compared with reference strains deposited in GenBank using BLAST. Subsequently, the NCBI ORF Finder was employed to identify ORFs within each gene segment. Sequence identity analysis was conducted using the MegAlign module in the DNASTAR 7.1 software package. For phylogenetic analysis, the nucleotide sequences of each RVB gene segment were subjected to a neighbor-joining algorithm implemented in MEGA 7.0 software [41]. The best-fit substitution models and 1000 bootstrap replicates were exploited to construct the phylogenetic trees, ensuring the reliability of the inferred evolutionary relationships.

## 3. Results

### 3.1. PoRVB Is the Primary Causative Agent in the Diarrhea Outbreaks of Newborn Piglets

All diarrheal samples were collected from newborn piglets in 3 farms that suffered severe diarrhea. RT-nPCR was performed to detect the presence of PEDV, TGEV, PDCoV, and PoRVA, but none of the samples were tested positive for these commonly prevalent porcine diarrhea-associated viruses. To investigate potential pathogens, MTT and MDA were conducted on pooled samples from each farm and the corresponding HTS data were analyzed for viral metagenomics. As a result, 779,190–1,236,941 reads were mapped to PoRVB in the pooled diarrheal samples, accounting for 99.44%−99.99% of the total mammalian virus reads. Additionally, PoRVA, PKV, and PAstV4 were identified in the samples from three farms. To further confirm the virus species present in the PoRVB-positive samples from each farm and obtain the whole genome sequences of the PoRVB strains, additional HTS (MTT and MDA) tests were carried out on 3–5 samples from each farm. The HTS data of the individual samples were consistent with those of the pooled samples, reaffirming PoRVB as the predominant virus in the diarrheic samples (Table 2). Compared with the viromic results of pooled samples, PoSaV was detected in one out of three samples from the AHLW farm, and PCV3 was found in two out of three samples from the JLCG farm. Based on the viromic data, the RPM of PoRVB in the pooled diarrheal samples ranged from 267,265.26 to 920,241.93 (Table 2), significantly higher than that of other identified viruses, including PoRVA, PKV, PAstV4, PoSaV, and PCV3. The presence of all mammalian viruses identified by HTS in individual samples from the three farms was validated by RT-nPCR and nPCR using the specific primers (Table 1). In the AHLW farm, the positive rates of PoRVB, PKV, and PoSaV were 83.3% (5/6), 16.7% (1/6), and 16.7% (1/6), respectively. In the JLCG farm, the virus-positive rates were PoRVB (100%, 8/8), PoRVA (0%, 0/8), PKV (100%, 8/8), and PCV3 (25%, 2/8), respectively. In the LNDC farm, the positive rates of PoRVB and PAstV4 were 90.9% (20/22) and 9.1% (2/22), respectively. Furthermore, RT-qPCR detection revealed PoRVB copy numbers ranged from 1159 to 271,833 per 10 ng RNA in 36 diarrhea samples across three farms. In the healthy and recovered piglets at the LNDC farm, PoRVB copy numbers ranged from 390 to 680 per 10 ng RNA (Table S2).

Moreover, the HTS data were analyzed with Metaphlan 4.0.6 software to explore the presence of bacteria in the diarrheal samples. Bacterial species such as *Escherichia coli* and *Enterococcus faecium* were identified. However, to the best of our knowledge, few of these bacteria have been reported to be major causative agents of diarrhea in piglets (Table S1). Additionally, no parasites were found in the HTS data. Taken together, PoRVB was identified as the primary causative agent for the outbreak of severe piglet diarrhea across these three distinct regions in China.

### 3.2. Genetic Characterization of the Identified PoRVB Strains

**Figure 1:**
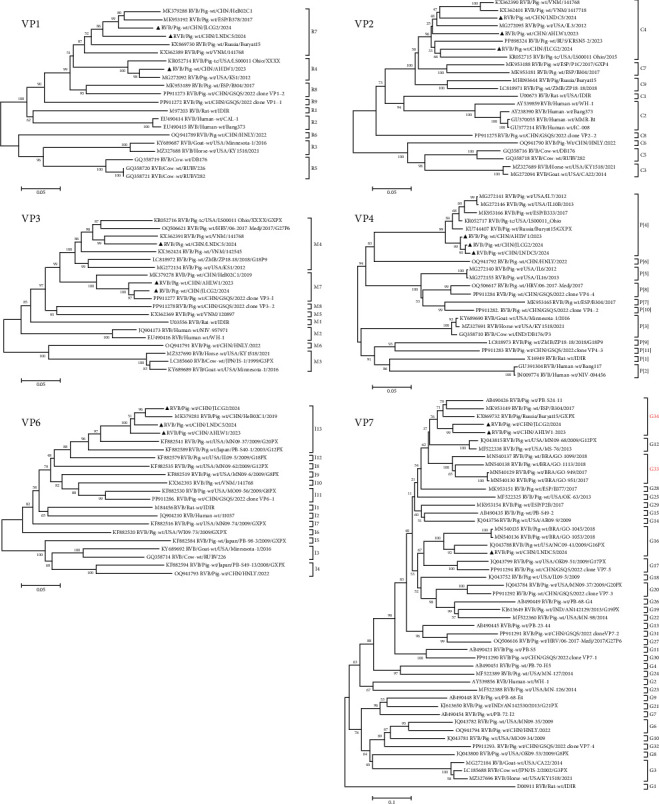
Phylogenetic trees constructed with the nucleotide sequences of PoRVB gene segments encoding VP1, VP2, VP3, VP4, VP6, and VP7. Phylogenetic analysis with the nucleotide sequences of PoRVB structural protein genes were conducted using the neighbor-joining method with 1000 bootstrap replicates. Scale bars indicate the number of substitutions per site. PoRVB strains JLCG2/2024, LNDC5/2024, and AHLW1/2023 identified in this study were highlighted in black triangles, and the new PoRVB genotypes classified in this study are marked in red. The best-fitting substitution models for phylogenetic analysis of the gene segments are different: VP1, VP2, VP3, VP4, and VP6, GTR + G + I; VP7, GTR + G. Bootstrap values are shown above branches to the left of major nodes.

**Figure 2:**
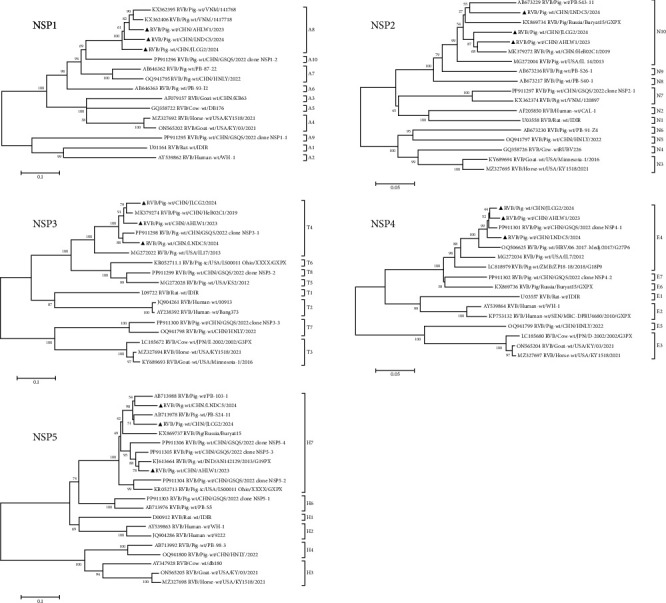
Phylogenetic trees constructed with the nucleotide sequences of PoRVB gene segments encoding NSP1–NSP5. Phylogenetic analysis, which was based on the nucleotide sequences of different gene fragments of PoRVB strains JLCG2/2024, LNDC5/2024, and AHLW1/2023 and the reference strains, was conducted using the neighbor-joining method with 1000 bootstrap repeats. PoRVB strains identified in this study are identified with black triangles. The best-fitting substitution models for phylogenetic analysis of the gene segments are different: NSP1, GTR + G; NSP3, HKY + G; NSP4, TN93 + G; NSP2 and NSP5, TN93 + G + I. Bootstrap values are shown above branches to the left of major nodes.

The whole genome sequences of PoRVB strains AHLW1/2023, JLCG2/2024, and LNDC5/2024 were obtained through *de novo* assembly of the PoRVB viral reads from individual samples. These have been deposited in GenBank under accession numbers PV893589–PV893621. All ORFs within the gene segments of these strains exhibited identical sizes to those of the reference strains (Table 3). However, sequence variations were observed in the nontranslated regions located at the termini of each gene segment. The three PoRVB strains identified in the present study shared 71.2%−95.6% nucleotide and 74.3%−99.0% amino acid sequence identities with each other. When compared to the most similar reference strains retrieved from GenBank, they showed 81.8%−95.6% nucleotide and 88.9%−99.0% amino acid sequence identities. The genotype constellation of AHLW1/2023 was assigned to be G34-P[4]-I13-R4-C4-M7-A8-N10-T4-E4-H7. It differed from JLCG2/2024 and LNDC5/2024 in one (R7) and three genotypes (G16, R7, and M4), respectively (Figures 1 and 2), highlighting both the genetic similarities and heterogeneities among the identified PoRVB strains.

Further compassion with the pathogenic Russian PoRVB strain Buryat15, which caused severe diarrhea in suckling piglets [20], revealed that the genotypes of gene segments VP1 (R7 in JLCG2/2024 and LNDC5/2024), VP3 (M4 in LNDC5/2024), VP4 (P[4]), VP6 (I13), VP7 (G34 in AHLW1/2023 and JLCG2/2024), NSP1 (A8), NSP2 (N10), NSP3 (T4), and NSP5 (H7) were identical to those of the PoRVB strains identified in this study. These segments shared 80.1%−87.0% sequence identities, suggesting a relatively close genetic relationship, despite significant divergence in the VP2 and NSP4 gene segments (76.5%−79.3%). For the pathogenic Brazilian strains [26], only the VP7 gene segment sequences were available. These strains were categorized into G12, G16, and G20, with G16 being the dominant genotype. In this study, four Brazilian PoRVB strains (GO-1099/1113/949/951), originally classified as G12 genotype, were reclassified as G33 based on the 80% genotyping cut-off value, as they shared 77.0%−80.3% nucleotide identities with the G12 reference strains. As depicted in Figure 1, LNDC5/2024 clustered with the Brazilian G16 strains, while the other two strains showed 80.2%−83.3% nucleotide identities of the VP7 gene to Eurasian strains (PB-S24-11, B304, and Buryat15). These two strains belonged to a new genotype, G34, and exhibited 76.4%−80.2% nucleotide identities to the G12 reference strains.

As shown in Table 3, the strains most similar to the different gene segments of the identified PoRVB strains originated from various countries. For example, the strains most closely related to the AHLW1/2023 strain, with 81.8%−94.3% nucleotide sequence identities, were from China, the USA, Vietnam, Russia, and India. For the JLCG2/2024 strain, the closest strains, showing 82.2%−95.6% nucleotide sequence identities, originated from China, Spain, the USA, Russia, Vietnam, and Japan. For the LNDC5/2024 strain, the closest strains, with 81.8%−92.9% nucleotide sequence identity, originated from China, the USA, Vietnam, and Japan. These data suggest that these pathogenic PoRVB strains may have originated from the reassortment events involving strains from different geographical regions.

## 4. Discussion

Since 2010, enteric viruses causing piglet diarrhea have inflicted substantial annual economic losses in China. Among these, the highly pathogenic PEDV and the recently emerging PoRVA [42–44] have been particularly concerning. In contrast, other diarrhea-associated viruses, such as PoRVB, have often been overlooked during routine diarrhea-associated virus detection, likely due to their relatively low prevalence, high genetic diversity and undetermined virulence. In this study, diarrheic samples collected from the farms experiencing large-scale piglet diarrhea were initially tested negative for major diarrheal viruses, including PEDV, TGEV, PDCoV, and PoRVA. However, surprisingly, PoRVB strains were identified in pooled or individual diarrheic samples using HTS. Several lines of evidence suggest that PoRVB could be the primary causative agent of these piglet diarrhea outbreaks: (1) Among all viruses detected by HTS in the diarrheic samples, PoRVB exhibited the highest viral abundance, far surpassing other viruses (PKV, PAstV4, PoSaV, and PCV3). Additionally, the relationship between the other viruses and the occurrence of diarrhea remains unclear except for PoSaV [45–47]. To date, there have been no reports of these other viruses causing diarrhea in pigs through infection alone. Furthermore, the positive rate and viral abundance of PoSaV were extremely low in the AHLW farm. Although PoSaV abundance was high in the LNDC farm, it was detected only in recovered pigs. This may be due to its low pathogenicity. These findings suggest that PoRVB-coinfected viruses play minimal roles in the large-scale piglet diarrhea outbreaks across the three farms. (2) PoRVB was detected in nearly all diarrheic samples from the farms with severe piglet diarrhea, with detection rates ranging from 83.3% to 100% across the sampled farms. RT-qPCR results revealed a significant disparity in viral load between healthy/recovered and diarrheic piglets, with a difference of 1–3 orders of magnitude per 10 ng of RNA copies. (3) Metaphlan 4.0.6 analysis detected multiple bacterial species but only identified *Escherichia coli* as the sole potential diarrhea-causing bacterium. *Escherichia coli* was the dominant species in some diarrhea-affected piglet samples and in recovered piglets from the LNDC farm (Table S1). However, the high relatively abundance of *Escherichia coli* (95.15%) in recovered piglets strongly indicates it functions as a commensal component of the gut microbiota rather than a pathogenic strain. These findings indicate that *Escherichia coli* is unlikely to be the causative agent of the three outbreaks. Furthermore, diarrhea-causing parasites were absent in the piglets, indicating that the episodes of diarrhea are not significantly associated with either bacterial or parasitic infections. (4) In recent years, sporadic reports have documented large-scale diarrhea outbreaks in newborn piglets caused by PoRVB reported in Russia, China, and Brazil [20, 25, 26]. Notably, the Russian strain RVB/Pig-wt/Russia/Buryat15 shared a relatively close genetic relationship with the PoRVB strains characterized in this study across most gene segments. Collectively, these findings strongly indicate that PoRVB is the primary etiological agent responsible for severe piglet diarrhea outbreaks.

Although the above evidence supports PoRVB as the primary pathogen, several limitations remain in this study. First, our attempts to isolate PoRVB from positive diarrhea samples were unsuccessful. To date, no successful PoRVB isolation has been reported globally, indicating that it may possess strict host cell tropism or necessitate specific conditions for replication. Future studies should focus on optimizing isolation systems and developing PoRVB reverse genetics systems to address isolation bottlenecks, which would enable direct pathogenicity testing and advance subsequent research. Additionally, the limited sample size at the AHLW farm imposed certain limitations (only six fecal samples were collected from diarrheic piglets; Section 2.1). This restriction may limit the ability to draw definitive conclusions about the true prevalence of PoRVB or the patterns of mixed infections. For example, only one sample from the AHLW farm tested positive for PKV/PoSaV. Future studies should expand sampling scope, such as collecting >10 diarrhea samples per farm covering multiple age groups and healthy/recovered piglets, to validate these findings.

The identification of PoRVB in three large-scale diarrhea outbreaks in this study also supports the idea that PoRVB prevalence in Chinese pig herds may be underestimated through comparison with recent reports. In China, PoRVB strains have recently been detected in over 10 provinces or regions, such as Guangxi, Chongqing, Guizhou, Heilongjiang, Shanxi, and Shandong [48]. However, only one case of piglet diarrhea associated with PoRVB has been reported, which was associated with a reassortant PoRVB strain named HNLY-2022. Genetic evidence indicates that this strain likely originated from a recombination event between herbivore and PoRVB lineages [25]. Further comparative analysis has revealed a significant divergence between the HNLY-2022 strain and the strains identified in this study. The nucleotide sequence identities range from 51.2% to 72.8%, and the amino acid identities range from 32.7% to 72.5%. This indicates substantial genetic differences and distinct origins between these strains. Moreover, genetic differences were also observed among the characterized PoRVB strains in this study. The genetic distances for all gene segments varied from 4.4% to 28.8%. The AHLW1/2023 and JLCG2/2024 strains are closely related and share identical genotypes for 10 gene segments, but they are genetically distant from the LNDC5/2024 strain. In addition, VP7 genotyping showed that AHLW1/2023 and JLCG2/2024 strains belong to the new genotype G34, which is neighboring to the clade of G12 in the phylogenetic tree (Figure 1), indicating a close evolutionary relationship and potential common ancestral origin of these two genotypes. To date, both G34 and G12 strains have only been detected in pigs, suggesting a possible pig-specific host range (pending further cross-species validation). For pathogenicity, besides the G34 strain in this study that caused large-scale diarrhea outbreak of piglets, previous report also indicated that G34 strain Buryat15 induced severe diarrhea and piglet death [24]. Thus, intensified surveillance should be conducted about the circulation and pathogenicity of PoRVB G34 strains.

Given that China is the world's largest pig-producing country and there has been relatively little attention paid to this pathogen, the identified PoRVB-associated diarrheal epidemics may merely represent the tip of an iceberg. It is highly probable that a considerable proportion of piglet diarrhea cases caused by PoRVB remain unrecognized or undiagnosed. Therefore, significant efforts should be made in order to effectively prevent and control PoRVB-associated diarrhea: (1) Precision surveillance of PoRVB in the pig industries in China and other countries. Considering the genetic diversity of PoRVB strains, the development of species-universal and accurate detection methods based on existing PoRVB sequences should be conducted for the routine diagnosis of diarrhea-associated viruses, such as RT-nPCR and real-time qPCR. Particularly, for the unexplained clinical diarrhea cases negative for the common viruses, the samples should be further analyzed via HTS. (2) Overcome the significant challenge of *in vitro* virus adaptation. This is essential for investigating the etiological properties of PoRVB and for establishing a challenge model to assess the viral virulence and the efficacy of vaccines. (3) Develop VP8*⁣*^*∗*^-based subunit vaccine against viruses of dominant genotypes. The VP8*⁣*^*∗*^ protein is a major protective antigen of RVs and has the ability to induce neutralizing antibodies against both homotypic and heterotypic viruses, laying a foundation for VP8*⁣*^*∗*^-based vaccine development. Sequence analysis of the three PoRVB strains in our study showed that the VP8*⁣*^*∗*^ domain shares 93.7%−94.9% amino acid homology. This high antigenic conservation implies stable antigenic characteristics of VP8*⁣*^*∗*^ among PoRVB strains. Thus, it can be considered an excellent candidate for the development of subunit vaccines.

## 5. Conclusion

The study has further confirmed that PoRVB is a significant enteric pathogen capable of inducing severe diarrhea in neonatal piglets. This finding serves as an early alert, emphasizing that during surveillance, prevention, and control of porcine viral diarrhea, this pathogen should not be overlooked.

## Figures and Tables

**Table 1:** Primers for virus detection.

Primer	Primer sequence (5′→3′)	Product size (bp)
JLCG-RVB-VP6-outF	GATTTGGGTGTGCATTTAGACAGAT	531
JLCG -RVB-VP6-outR	ATTGAGCAAGTTGTAAATAGGCCAC	
JLCG -RVB-VP6-inF	AAATCTCATGCGGTTACACATCG	263
JLCG -RVB-VP6-inR	TCAGGAATGGATTCTGAGCATAGG	
LNDC-RVB-VP6-outF	TGACTGGTGGAAATATCAGAGCTAC	458
LNDC-RVB-VP6-outR	AGTGTCGCGAGTTTCAAACATAAAG	
LNDC-RVB-VP6-inF	GGATTCCGAGCATAGATTCATAGTT	305
LNDC-RVB-VP6-inR	CTGTTCCTAGACCTTCTTGATCTTT	
AHLW-RVB-VP6-outF	TCACATCCCCAATTTCAGTTGCAAA	470
AHLW-RVB-VP6-outR	GATTCTAAATCTCATGCGGTTACAC	
AHLW-RVB-VP6-inF	GTTATCGAGCAAGTTGTGAACAGAC	331
AHLW-RVB-VP6-inR	CAACTCCAGTCTCGGTTTGAATTAT	
JLCG-PCV3-outF	AGCAATTCATCAAATGGAACCCAC	912
JLCG-PCV3-outR	TAGCTCTGTGTCTCATTTTGGTGC	
JLCG-PCV3-inF	TTCAGTCAGATTTCCCGACGTCTC	560
JLCG-PCV3-inR	GTAGGGAGAAAAAGTGGTATCCCAT	
JLCG-RVA-VP6-outF	TGGAGGTTCTGTACTCATTGTCAAA	345
JLCG-RVA-VP6-outR	GTGCTTCAGATTGTGGTGCTATTCC	
JLCG-RVA-VP6-inF	GGATGCTAGAGATAAAATCGTTGAAGG	272
JLCG-RVA-VP6-inR	CTCTAGCTATCTCATCCATACATAC	
JLCG-PKV-outF	CACTTTGTTTCTAACATCACYGGTG	829
JLCG-PKV-inF	ACTCCATTGAGACTACAGCCACA	639
JLCG-PKV-R	TTCGCTTTGCCACGAATCCATGT	
AHLW-SAV-outF	GCTGAAAAGTGCCTGTTGATTTGTT	540
AHLW-SAV-inF	TTAAGCAGGGTGAACCCAAAATCTG	368
AHLW-SAV-R	CAGGACTGAACCCCTACACACTT	
AHLW-PKV-outF	AGGGAATGGAAATAGGAATGGTAGC	449
AHLW-PKV-outR	CATTGAATCTGGTTCTGCTACTACT	
AHLW-PKV-inF	GGTGGAGCAAAGTAAATAGTTATGC	300
AHLW-PKV-inR	CCGTACCACTTTCTCCTATACTGATA	
LNDC-PAstV4-outF	CTAACATTTTGGGATAAGCTGGTGT	549
LNDC-PAstV4-outR	CAACACCTTAAATGTGATGATCCAC	
LNDC-PAstV4-inF	GCACTCAGGATAGTCCTTTTCAAAA	329
LNDC-PAstV4-inR	CTTGAATACCAGCAGAGGATGAAGC	

**Table 2:** Virus species and their abundance in the mixed and individual samples from each sampled farm^a^.

Virus species							
Sample	PoRVA	PoRVB	PKV	PAstV4	PoSaV	Porcine bastrovirus	PCV3
AHLW1	–	625,593.58	43.45	–	3.47	–	–
AHLW2	–	590,640.64	–	–	–	–	–
AHLW3	–	366,576.97	–	–	–	–	–
AHLW-pooled	–	911,408.16	1.61	–	–	–	–
JLCG1	538.73	362,697.75	18,804.63	–	–	–	124.51
JLCG2	–	458,726.60	12,939.24	–	–	–	–
JLCG3	–	920,241.93	486.77	–	–	–	4.25
JLCG-pooled	338.35	804,540.47	776.28	–	–	–	–
LNDC1	–	287,587.01	–	–	–	–	–
LNDC2	–	382,801.86	–	–	–	–	–
LNDC3	–	267,265.26	–	1563.75	–	–	–
LNDC4	–	843,976.38	–	–	–	–	–
LNDC5	–	851,361.99	–	–	–	–	–
LNDC-diarrhea-pooled	–	902,155.01	–	49.65	–	–	–
LNDC-healthy-pooled	–	699.41	–	–	–	348.91	–
LNDC-recovered-pooled	–	10,490.54	–	–	170,910.04	–	–

*Note: “*–” indicates “the target index was not detected in the sample.”

^a^RPM represents viral abundance.

**Table 3:** Percentage of nucleotide and amino acid identities between AHLW1/2023, JLCG2/2024, LNDC5/2024, and their most similar RVB strains.

Sample	Segment	Gene	Length		Accession number	Most similar strain	nt (%)	aa (%)
			Gene/ORF					
AHLW1/2023	1	VP1	3538	3483	MG272092	RVB/Pig-wt/USA/KS1/2012	94.0	97.3
	2	VP2	2853	2805	KX362401	RVB/Pig-wt/VNM/14177_18	84.2	95.9
	3	VP3	2367	2292	PP911277.1	RVB/Pig-wt/CHN/GSQS/2022 VP3-1	93.4	96.4
	4	VP4	2315	2250	KR052717	RVB/Pig-tc/USA/LS00011_Ohio	82.2	90.5
	6	VP6	1285	1176	MK379281	RVB/Pig-wt/CHN/HeB02_C1	89.1	96.1
	9	VP7	818	747	KX869732	RVB/Pig-wt/Russia/Buryat15	81.8	91.2
	5	NSP1-1	1305	963	KX362406	RVB/Pig-wt/VNM/14177_18	93.6	96.6
	5	NSP1-2	1305	306	KX362406	RVB/Pig-wt/VNM/14177_18	94.3	98.0
	7	NSP2	1032	906	MK379272	RVB/Pig-wt/CHN/HeB02_C1	90.6	97.0
	8	NSP3	986	828	MK379274	RVB/Pig-wt/CHN/HeB02_C1	92.7	92.5
	10	NSP4	769	666	PP911301	RVB/Pig-wt/CHN/GSQS/2022 clone NSP4-1	94.1	94.1
	11	NSP5	674	525	KJ613664	RVB/Pig-wt/IND/AN142129/2013	94.3	94.9

JLCG2/2024	1	VP1	3526	3483	MK953192	RVB/Pig-wt/ESP/B378/2017/GXP[X]	87.9	94.6
	2	VP2	2864	2805	PP898324	RVB/Pig-wt/RUS/KRSN5-2/2023	83.4	92.3
	3	VP3	2356	2292	PP911277	RVB/Pig-wt/CHN/GSQS/2022 clone VP3-1	94.9	97.2
	4	VP4	2299	2250	MG272141	RVB/Pig-wt/USA/IL7/2012	82.2	89.2
	6	VP6	1269	1176	MK379281	RVB/Pig-wt/CHN/HeB02_C1	95.2	95.8
	9	VP7	830	747	KX869732	RVB/Pig-wt/Russia/Buryat15	84.4	90.8
	5	NSP1-1	1284	963	KX362406	RVB/Pig-wt/VNM/14177_18	90.3	95.6
	5	NSP1-2	1284	306	KX362406	RVB/Pig-wt/VNM/14177_18	94.3	99.0
	7	NSP2	1038	906	MK379272	RVB/Pig-wt/CHN/HeB02_C1	89.1	96.7
	8	NSP3	984	828	MK379274	RVB/Pig-wt/CHN/HeB02_C1	93.5	94.0
	10	NSP4	769	666	PP911301	RVB/Pig-wt/CHN/GSQS/2022 clone NSP4-1	95.6	96.4
	11	NSP5	640	525	AB713978	RVB/pig-wt/JPN/PB-S24-11/2013	89.5	93.7

LNDC5/2024	1	VP1	3524	3483	MK379288	RVB/Pig-wt/CHN/HeB02_C1	84.2	93.3
	2	VP2	2860	2805	KX362390	RVB/Pig-wt/VNM/14176_8	84.9	95.1
	3	VP3	2365	2292	KX362391	RVB/Pig-wt/VNM/14176_8	84.6	91.8
	4	VP4	2303	2250	MG272141	RVB/Pig-wt/USA/IL7/2012	81.8	88.9
	6	VP6	1282	1176	MK379281	RVB/Pig-wt/CHN/HeB02_C1	90.7	97.5
	9	VP7	814	747	JQ043788	RVB/Pig-wt/USA/NC09-41/2009/G16P[X]	87.4	90.7
	5	NSP1-1	1284	963	KX362406	RVB/Pig-wt/VNM/14177_18	90.2	95.6
	5	NSP1-2	1284	306	KX362406	RVB/Pig-wt/VNM/14177_18	92.4	96.0
	7	NSP2	1037	906	AB673229	RVB/pig-wt/JPN/PB-S43-11	87.3	96.3
	8	NSP3	971	828	PP911298	RVB/Pig-wt/CHN/GSQS/2022 clone NSP3-1	92.9	93.3
	10	NSP4	768	666	PP911301	RVB/Pig-wt/CHN/GSQS/2022 clone NSP4-1	93.1	94.1
	11	NSP5	727	525	AB713988	RVB/Pig-wt/JPN/PB-103-1	89.7	92.6

## Data Availability

The data that support the findings of this study are available in the supporting information of this article.

## Supporting Information

Supporting Information Table S1. Relative abundance of bacteria and total number of reads per sample. Table S2. Viral loads of PoRVB in the anal swab samples of piglets from three farms.
