# Supplementary material for: Severe Diarrhea Outbreaks in Newborn Piglets in China Associated With Porcine Rotavirus B
Source: Transbound Emerg Dis. 2025 Nov 21;2025:5588912. doi: 10.1155/tbed/5588912 (PMC12662673; doi:10.1155/tbed/5588912)
Supplement: Supporting Information — Table S1. Relative abundance of bacteria and total number of reads per sample. Table S2. Viral loads of PoRVB in the anal swab samples of piglets from three farms. [file 5588912.f1.docx]

| Supplementary Table 1 Relative abundance of bacteria and total number of reads per sample | | | | | | | | | | | | | | | | |
| --- | --- | --- | --- | --- | --- | --- | --- | --- | --- | --- | --- | --- | --- | --- | --- | --- |
| Relative abundance (%) | AHLW1 | AHLW2 | AHLW3 | AHLW-pooled | JLCG1 | JLCG2 | JLCG3 | JLCG-pooled | LNDC1 | LNDC2 | LNDC3 | LNDC4 | LNDC5 | LNDC- diarrhea -pooled | LNDC- healthy -pooled | LNDC- recovered -pooled |
| *Veillonella caviae* | 31.83 | - | 17.44 | 6.19 | 1.92 | 3.24 | 5.59 | 2.61 | - | - | - | 12.90 | 10.91 | 1.19 | 1.32 | - |
| *Lactobacillus amylovorus* | 19.26 | 3.60 | - | - | - | - | - | - | - | - | - | - | - | - | - | - |
| *Phocaeicola vulgatus* | 9.00 | 18.98 | 1.36 | 9.26 | - | - | - | - | - | - | - | 6.45 | 2.87 | 2.61 | - | - |
| *Bacteroides fluxus* | 7.78 | - | - | 3.86 | - | - | - | - | - | - | - | - | - | - | - | - |
| *Escherichia coli* | 6.58 | 28.86 | 4.22 | - | 44.74 | 3.16 | 10.76 | 7.77 | 45.58 | 97.94 | 93.11 | 63.37 | 30.33 | 62.53 | 18.84 | 95.15 |
| *Lactobacillus johnsonii* | 4.57 | - | 6.78 | 1.44 | 13.62 | - | - | - | 3.48 | - | - | 6.27 | 5.79 | - | - | - |
| *Actinobacillus porcinus* | 3.26 | - | - | - | - | - | - | - | - | - | - | - | - | - | - | - |
| *Butyricicoccus intestinisimiae* | 2.51 | - | - | - | - | - | - | - | - | - | - | - | - | - | - | - |
| *Bacteroides fragilis* | 1.95 | - | - | 13.66 | - | 16.96 | - | 15.81 | - | - | - | - | - | 1.52 | 55.32 | - |
| *Lactobacillus delbrueckii* | 1.64 | - | 1.22 | 8.79 | - | 3.48 | - | 4.48 | - | - | - | - | 7.35 | - | - | - |
| *Limosilactobacillus mucosae* | 1.53 | - | 9.34 | - | 3.95 | 6.42 | 2.13 | - | - | - | - | - | - | - | - | - |
| *Erysipelatoclostridium ramosum* | 1.33 | - | - | - | - | - | 6.08 | - | - | - | - | - | - | - | - | - |
| *Faecalimonas umbilicata* | - | 5.66 | - | - | - | - | 17.76 | - | - | - | - | - | - | - | - | - |
| *Enterococcus faecium* | - | 5.45 | - | - | - | - | - | - | 50.49 | 2.04 | 6.26 | 3.72 | - | 22.14 | 1.6 | - |
| *Cupriavidus metallidurans* | - | 3.22 | - | - | - | - | - | - | - | - | - | - | - | - | - | - |
| GGB25063 SGB36986 | - | 1.98 | - | - | 4.84 | - | 19.11 | - | - | - | - | - | - | - | - | - |
| *Fusobacterium mortiferum* | - | 1.69 | - | 1.36 | - | - | - | 5.21 | - | - | - | - | - | - | - | - |
| *Anaeromassilibacillus sp* An172 | - | - | 7.03 | - | 1.61 | - | 4.20 | - | - | - | - | - | 3.73 | - | - | - |
| GGB9675 SGB15175 | - | - | 6.35 | - | - | - | - | - | - | - | - | - | - | - | - | - |
| *Phascolarctobacterium succinatutens* | - | - | 4.79 | - | - | - | - | - | - | - | - | - | - | - | - | - |
| GGB13463 SGB15285 | - | - | 4.56 | - | - | - | - | - | - | - | - | - | - | - | - | - |
| *Faecalicatena contorta* | - | - | 4.32 | - | - | - | - | - | - | - | - | - | - | - | - | - |
| *Mogibacterium kristiansenii* | - | - | 3.74 | - | - | - | - | - | - | - | - | - | - | - | - | - |
| GGB9747 SGB15355 | - | - | 3.00 | - | - | - | - | - | - | - | - | - | - | - | - | - |
| *Rothia endophytica* | - | - | 2.82 | - | - | - | - | - | - | - | - | - | - | - | - | - |
| *Weissella cibaria* | - | - | 1.87 | - | - | - | - | - | - | - | - | - | - | - | - | - |
| *Clostridium scindens* | - | - | 1.50 | - | 6.79 | - | 2.20 | - | - | - | - | - | - | - | - | - |
| *Catenibacterium mitsuokai* | - | - | 1.35 | - | - | - | - | - | - | - | - | - | - | - | - | - |
| GGB9642 SGB15119 | - | - | 1.28 | - | - | - | - | - | - | - | - | - | - | - | - | - |
| *Streptococcus orisratti* | - | - | 1.17 | - | - | - | - | - | - | - | - | - | - | - | - | - |
| GGB4884 SGB6823 | - | - | 1.04 | - | - | - | - | - | - | - | - | - | - | - | - | - |
| *Prevotella stercorea* | - | - | - | 21.24 | - | - | - | - | - | - | - | - | - | - | - | - |
| *Sphingomonas sp* 3F27F9 | - | - | - | 6.96 | - | - | - | - | - | - | - | - | - | - | - | - |
| *Prevotella pectinovora* | - | - | - | 5.63 | - | - | - | - | - | - | - | - | - | - | - | - |
| GGB32782 SGB48200 | - | - | - | 5.02 | - | - | - | - | - | - | - | - | - | - | - | - |
| *Bacteroides pyogenes* | - | - | - | 4.18 | - | - | - | - | - | - | - | - | - | - | - | - |
| *Prevotellamassilia timonensis* | - | - | - | 2.99 | - | - | - | - | - | - | - | - | - | - | - | - |
| GGB74464 SGB48212 | - | - | - | 2.25 | - | - | - | - | - | - | - | - | - | - | - | - |
| *Phocaeicola coprophilus* | - | - | - | 1.43 | - | - | - | - | - | - | - | - | - | - | - | - |
| GGB34009 SGB53482 | - | - | - | 1.41 | - | - | - | - | - | - | - | - | - | - | - | - |
| *Bacteroides thetaiotaomicron* | - | - | - | 1.12 | - | - | - | 1.59 | - | - | - | - | - | - | - | - |
| *Eisenbergiella massiliensis* | - | - | - | - | 8.29 | - | - | - | - | - | - | - | - | - | - | - |
| *Ligilactobacillus salivarius* | - | - | - | - | 3.91 | 3.04 | 4.86 | 54.29 | - | - | - | - | 2.92 | - | - | - |
| *Actinobacillus minor* | - | - | - | - | 1.76 | - | - | - | - | - | - | - | - | - | - | - |
| *Limosilactobacillus reuteri* | - | - | - | - | 1.59 | - | - | - | - | - | - | 1.33 | - | - | - | - |
| *Bacteroides xylanisolvens* | - | - | - | - | 1.42 | - | - | - | - | - | - | - | - | - | - | - |
| *Eggerthella lenta* | - | - | - | - | 1.18 | - | - | - | - | - | - | - | - | - | - | - |
| *Limosilactobacillus vaginalis* | - | - | - | - | - | - | 11.74 | - | - | - | - | - | - | - | - | - |
| *Ruminococcus gnavus* | - | - | - | - | - | - | 8.18 | - | - | - | - | - | - | - | 12.51 | - |
| *Blautia producta* | - | - | - | - | - | - | 3.58 | - | - | - | - | - | - | - | - | - |
| *Phocaeicola plebeius* | - | - | - | - | - | - |  | 3.04 | - | - | - | - | - | - | - | - |
| *Streptococcus gallolyticus* | - | - | - | - | - | 62.44 | - | - | - | - | - | - | 8.56 | 5.18 | - | - |
| *Lactobacillus crispatus* | - | - | - | - | - | - | - | - | - | - | - | - | 14.05 | 1.73 | - | - |
| *Clostridium symbiosum* | - | - | - | - | - | - | - | - | - | - | - | - | - | 1.26 | - | - |
| *Holdemanella porci* | - | - | - | - | - | - | - | - | - | - | - | - | 10.40 | - | - | - |
| *Oliverpabstia intestinalis* | - | - | - | - | - | - | - | - | - | - | - | - | 3.24 | - | - | - |
| *Butyricicoccus* SGB14985 | - | - | - | - | - | - | - | - | - | - | - | - | - | - | 3.28 | - |
| *Enterococcus gallinarum* | - | - | - | - | - | - | - | - | - | - | - | - | - | - | 1.82 | - |
| *Enterococcus hirae* | - | - | - | - | - | - | - | - | - | - | - | - | - | - | 1.18 | - |
| *Enterococcus avium* | - | - | - | - | - | - | - | - | - | - | - | - | - | - | 1.12 | - |
| GGB1627 SGB2227 | - | - | - | - | - | - | - | - | - | - | - | - | - | - | - | 2.72 |
| Total number of bacterial reads | 3640162 | 601289 | 1462770 | 287059 | 3702368 | 215606 | 1144544 | 246804 | 299787 | 1206318 | 6641363 | 1921390 | 632886 | 903273 | 1248715 | 3723928 |

"-" indicates the target index was not detected in the sample

| Sample | Healthy status | PoRVB copy numbers per 10 ng RNA |
| --- | --- | --- |
| AHLW1 | diarrhea | 21167 |
| AHLW2 | diarrhea | 6208 |
| AHLW3 | diarrhea | 2832 |
| AHLW4 | diarrhea | 8112 |
| AHLW5 | diarrhea | 1890 |
| AHLW6 | diarrhea | 10598 |
| JLCG1 | diarrhea | 21167 |
| JLCG2 | diarrhea | 93333 |
| JLCG3 | diarrhea | 6597 |
| JLCG4 | diarrhea | 25483 |
| JLCG5 | diarrhea | 1910 |
| JLCG6 | diarrhea | 5037 |
| JLCG7 | diarrhea | 2240 |
| JLCG8 | diarrhea | 9673 |
| LNDC1 | diarrhea | 4377 |
| LNDC2 | diarrhea | 6790 |
| LNDC3 | diarrhea | 1545 |
| LNDC4 | diarrhea | 82783 |
| LNDC5 | diarrhea | 49967 |
| LNDC6 | diarrhea | 36433 |
| LNDC7 | diarrhea | 42517 |
| LNDC8 | diarrhea | 46850 |
| LNDC9 | diarrhea | 33283 |
| LNDC10 | diarrhea | 2253 |
| LNDC11 | diarrhea | 271833 |
| LNDC12 | diarrhea | 104983 |
| LNDC13 | diarrhea | 262500 |
| LNDC14 | diarrhea | 76683 |
| LNDC15 | diarrhea | 29267 |
| LNDC16 | diarrhea | 75550 |
| LNDC17 | diarrhea | 8172 |
| LNDC18 | diarrhea | 58917 |
| LNDC19 | diarrhea | 40000 |
| LNDC21 | diarrhea | 8922 |
| LNDC22 | diarrhea | 2605 |
| LNDC23 | healthy | 1159 |
| LNDC24 | healthy | 421 |
| LNDC25 | healthy | 680 |
| LNDC26 | healthy | 459 |
| LNDC27 | healthy | 559 |
| LNDC28 | recovered | 390 |
| LNDC29 | recovered | 543 |

Supplementary Table 2 Viral loads of PoRVB in the anal swab samples of piglets from three farms
